# Cytokines in clinical cancer immunotherapy

**DOI:** 10.1038/s41416-018-0328-y

**Published:** 2018-11-09

**Authors:** Pedro Berraondo, Miguel F. Sanmamed, María C Ochoa, Iñaki Etxeberria, Maria A. Aznar, José Luis Pérez-Gracia, María E. Rodríguez-Ruiz, Mariano Ponz-Sarvise, Eduardo Castañón, Ignacio Melero

**Affiliations:** 1 Immunology and Immunotherapy Program, Center for Applied Medical Research, CIMA, University of Navarra, Pamplona, Spain; 2 Navarra Institute for Health Research (IDISNA), Pamplona, Spain; 3 Centro de Investigación Biomédica en Red de Cáncer (CIBERONC), Pamplona, Spain; 4 Department of Oncology and immunology, Clínica Universidad de Navarra, Pamplona, Spain

**Keywords:** Drug development, Cytokines, Immunotherapy

## Abstract

Cytokines are soluble proteins that mediate cell-to-cell communication. Based on the discovery of the potent anti-tumour activities of several pro-inflammatory cytokines in animal models, clinical research led to the approval of recombinant interferon-alpha and interleukin-2 for the treatment of several malignancies, even if efficacy was only modest. These early milestones in immunotherapy have been followed by the recent addition to clinical practice of antibodies that inhibit immune checkpoints, as well as chimeric antigen receptor T cells. A renewed interest in the anti-tumour properties of cytokines has led to an exponential increase in the number of clinical trials that explore the safety and efficacy of cytokine-based drugs, not only as single agents, but also in combination with other immunomodulatory drugs. These second-generation drugs under clinical development include known molecules with novel mechanisms of action, new targets, and fusion proteins that increase half-life and target cytokine activity to the tumour microenvironment or to the desired effector immune cells. In addition, the detrimental activity of immunosuppressive cytokines can be blocked by antagonistic antibodies, small molecules, cytokine traps or siRNAs. In this review, we provide an overview of the novel trends in the cytokine immunotherapy field that are yielding therapeutic agents for clinical trials.

## Introduction

Cytokines are polypeptides or glycoproteins with a molecular weight usually below 30 kDa that provide growth, differentiation and inflammatory or anti-inflammatory signals to different cell types. Cytokines are most often released during a defined period in response to a stimulus, and the extent of their action is short-lived due to their limited half-life in the circulation. As a result, cytokines normally exert an autocrine or paracrine effect. As an exception to the general rule, cytokines such as interleukin (IL)-7 or haematopoietic growth factors are produced homeostatically in a continuous fashion.

Cytokine target cells express high-affinity receptors on their cellular membrane. Following cytokine binding, the receptors trigger intracellular signalling which leads to modifications in gene transcription. Cytokines thereby modify proliferation and differentiation and induce or modify particular cell functions. Target cells expressing the corresponding sets of receptors integrate the information derived from the concentration and timing of exposure to different cytokines. Thus, synergy or antagonism among different cytokines is a common characteristic, with high degrees of complexity.

### Cytokines as a monotherapy

Several cytokines limit tumour cell growth by a direct anti-proliferative or pro-apoptotic activity, or indirectly by stimulating the cytotoxic activity of immune cells against tumour cells. A paradigmatic case is interferon-alpha (IFN-α), first discovered in 1957 as a result of its antiviral properties.^1^ After 13 years, Gresser and Bourali^2^ described the anti-tumour activity of IFN-α against different tumour cell lines inoculated in mice. This discovery of the ability of cytokines to potentiate immune responses against cancer in conjunction with the development of recombinant DNA technologies led, in the 1980s and 1990s, to intense preclinical and clinical investigation of the potential anti-tumour activity of several recombinant cytokines. However, results from clinical trials failed to meet the high expectations raised in preclinical models and highlighted the limitations of approaches based on unmodified recombinant proteins. These limitations include the short half-life of most cytokines and narrow therapeutic windows with only modest anti-tumour efficacy, at least as monotherapies. Only two cytokines, IL-2 and IFN-α, demonstrated mild clinical benefit and consequently received The Food and Drug Administration (FDA) approval for the treatment of several malignant diseases. IL-2 was approved for the treatment of advanced renal cell carcinoma (RCC)^3^ and metastatic melanoma,^4^ whereas IFN-α was approved for the treatment of hairy cell leukaemia,^5^ follicular non-Hodgkin lymphoma,^6^ melanoma^7^ and AIDS-related Kaposi’s sarcoma.^8^ The clinical use of these cytokines marked a milestone in cancer immunotherapy, as it was the first demonstration that immunotherapy could favourably tilt the balance between cancer and the anti-tumour immune response, leading to durable objective responses. However, the low response rate and high toxicity associated with high-dose IL-2 and IFN-α administration have relegated the clinical use of these cytokines in favour of targeted therapy and immune checkpoint inhibitors.^9,10^

### Potentiating the effects of immunotherapies

Immune checkpoint inhibitors represent a revolution in cancer immunotherapy. Clinical immunotherapy with monoclonal antibodies to block the CTLA-4 (cytotoxic T-lymphocyte-associated protein 4) or programmed cell death protein 1 (PD-1)–PD-1 ligand (PD-L1) axes have been FDA-approved for the treatment of several malignancies such as melanoma, non-small cell lung cancer, RCC, Hodgkin lymphoma, Merkel cell carcinoma, head and neck cancer and carcinoma of the bladder. These novel therapies have yielded long-lasting responses in a fraction of patients. In contrast to recombinant IL-2 or IFN-α as mentioned above, immune checkpoint inhibitors have a more favourable safety profile. Any adverse effects are mainly autoimmune-like syndromes that can usually be controlled by treatment with corticosteroids or other anti-inflammatory drugs, such as infliximab.^11^ The synergistic combination of anti-CTLA-4 and anti-PD-1 monoclonal antibodies has been approved for the treatment of advanced melanoma, microsatellite instability-high or mismatch repair-deficient metastatic colorectal cancer and advanced RCC by the FDA and has improved the overall response rate of patients.^12–14^ The clinical success of this combination provides the rationale for new combination approaches in immunotherapy.^15^ In this context, cytokines are being incorporated into combination clinical trials, mainly in conjunction with anti-PD-1 and anti-PD-L1 monoclonal antibodies.

Adoptive T cell therapies are also coming of age due to impressive efficacy results and FDA approval of anti-CD19 chimeric antigen receptor (CAR) T cells,^16^ and the use of cultures of tumour-infiltrating T lymphocytes (TILs).^17^ Notably, these approaches are totally dependent on cytokines for in vitro expansion and in vivo persistence of transferred T cells. These cellular immunotherapies can be optimised by the incorporation of cytokine genes into the lentiviral vector that encodes the CARs.^18^

The search for the next generation of cytokine-based drugs is based on three concepts. The first concept is synergistic combinations, such that the approved anti-PD-1–PD-L1 monoclonal antibodies and CAR19 T cells provide a suitable basis for combining with cytokines. The second concept looks at improved pharmacokinetics. The systemic administration of cytokines requires optimisation of the pharmacokinetic profile to increase the half-life in circulation (surpassing the kidney filtration threshold) and to increase the cytokine concentration in the tumour microenvironment (TME). This aim can be achieved by conjugating polyethylene glycol (PEG) to the cytokine or by constructing a fusion protein with antibodies, Fc domains, apolipoprotein A-I, albumin or the latent peptide of transforming growth factor-β (TGF-β). The third concept is that of local administration. An alternative strategy to achieve high local concentrations of cytokines in the TME is to directly inject the recombinant protein^19^ or intratumoural gene therapy vectors that encode the cytokine^20,21^ into the TME. Clinical trials are currently making use of oncolytic viruses, plasmid electroporation and intratumoural injection of lipid nanoparticles loaded with modified messenger RNAs.

In this review, we describe how these blueprint concepts are being applied to several cytokine-based compounds already undergoing clinical trials. Among the immunostimulatory cytokines that potentiate immune responses against cancer, we focus our review on IFN-α, the IL-2 family, IL-12, granulocyte-macrophage colony-stimulating factor (GM-CSF) and IL-10. In the case of immunosuppressive cytokines, we describe strategies to block the activity of tumour necrosis factor-alpha (TNF-α), TGF-β and cytokines that nurture tumour-associated myeloid cells, such as colony-stimulating factor-1 (CSF-1). Finally, we discuss the key concepts that must be considered for future development of cytokine-based immunotherapy.

## Potentiating the immune response

Pro-inflammatory cytokines can contribute to cancer immunotherapy, acting on every phase of the cancer immunity cycle.^22,23^ Thus, cytokines can improve antigen priming, increase the number of effector immune cells in the TME and enhance their cytolytic activity. However, making drugs based on cytokines requires fine-tuning of their pharmacological properties using biotechnological strategies.

### Interferon-α

Since the first approval of IFN-α for the treatment of hairy cell leukaemia in 1986, this family of cytokines has been used for the treatment of several haematological malignancies and solid tumours at high doses to exploit their direct pro-apoptotic/anti-proliferative activity on tumour cells. These high doses of IFN-α also exert anti-tumour activity due to effects on the tumour vasculature as IFN-α displays a potent antiangiogenic activity.^24^ PEGylated IFN-α was also approved for the adjuvant treatment of melanoma.^25^ This variant consists of a chemical modification that increases its half-life in circulation and therefore prolongs the exposure of the tumour cells to high IFN-α concentrations. However, the advent of targeted therapies and novel immunotherapies with superior safety and efficacy profiles has reduced clinical use of IFN-α in oncohaematology. This situation might be reverted with new cytokine modifications that exploit the immunostimulatory properties of IFN-α, as this cytokine is critical for the maturation of dendritic cells (DCs) and for the acquisition of effector function by T lymphocytes.^26^

One strategy to unplug the cytotoxic activity of IFN-α from its immunostimulatory activity involves its fusion to apolipoprotein A-I.^27^ The apolipoprotein A-I moiety incorporates the cytokine into high-density lipoproteins, improving the pharmacokinetics and anti-tumour activity of IFN-α.^28,29^ AcTakines, activity-on-Target cytokines, can also minimise the toxicity of IFN-α and maximise its immunostimulatory activity. This strategy is based on the fusion of a mutated cytokine that shows reduced affinity for its receptor to a cell-specific targeting domain. IFN-α fused to single domain antibodies targeting Clec9A, a molecule expressed on DCs specialised in cross-priming, displays a potent anti-tumour effect.^30^ Finally, IFN-α immunocytokines have been demonstrated to exert an anti-tumour effect mediated by the activation of immune system cells.^31,32^ In summary, type I IFN immunobiology is likely to be exploited in the near future but is not currently in the frontline oncology armamentarium.

### Interleukin-2

IL-2 is viewed as a key cytokine in promoting the expansion of natural killer (NK) cells and T lymphocytes. Thus, it is widely used in protocols of adoptive transfer for both expanding lymphocytes in culture and increasing the persistence of transferred cells in cancer patients. The infusion of this cytokine at high doses is currently approved for the treatment of metastatic RCC and metastatic melanoma. However, the systemic administration of this cytokine at the recommended dose is hampered by its toxic profile, which includes frequent grade 3 and 4 adverse effects. Second-generation IL-2-based therapies with improved pharmacokinetic and pharmacodynamic profiles are being developed. Improvement of the pharmacokinetic profile is achieved through covalent binding of IL-2 to moieties that increase the half-life in circulation, such as the Fc domains of immunoglobulins or PEG molecules, or by chimerisation with antibodies that target the cytokine to the TME. Improvement of the pharmacodynamic properties is attained by using biotechnology tricks to reduce binding to the high-affinity IL-2 receptor while maintaining binding to the medium-affinity IL-2 receptor to increase the amount of cytokine that is available to stimulate NK and T cells.

**Fig. 1:**
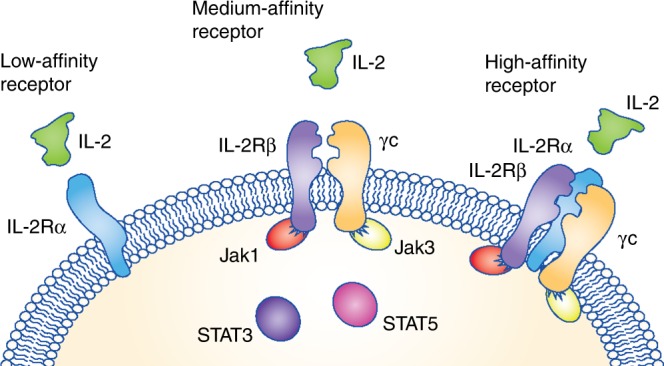
Interleukin (IL)-2 receptors. IL-2 is recognised by three types of receptor complex expressed on natural killer (NK) and T lymphocytes. The non-signalling, low-affinity receptor is composed of the IL-2Rα chain alone. The medium-affinity receptor is composed of the IL-2Rβ chain and the common γ chain. Finally, the high-affinity IL-2 receptor is composed of the IL-2Rα, the IL-2Rβ chain and the common γ chain. Ligand binding to the medium-affinity and high-affinity receptors leads to the phosphorylation of Janus kinase-1 (JAK1) and JAK3 and the recruitment and subsequent phosphorylation of signal transducer and activator of transcription-3 (STAT3) and STAT5, and ensuing transcriptional changes

The IL-2 receptor is composed of three different complexes formed by three chains (Fig. 1). The low-affinity receptor is composed of the IL-2Rα chain alone but does not trigger an intracellular signalling cascade. Thus, the two receptors that induce signalling are the medium-affinity and the high-affinity receptors. The medium-affinity receptor is composed of the IL-2Rβ chain and the common γ chain; when the IL-2Rα chain is also present in the receptor complex, IL-2 is bound with high affinity. IL-2Rα is highly expressed on T regulatory (Treg) cells and therefore the high-affinity IL-2 receptor skews IL-2 activity towards the expansion of Treg cells, while limiting the bioavailability of the cytokine to stimulate anti-tumour effector NK and T lymphocytes.^9^

Several of these second-generation IL-2-based compounds engineered to avoid binding to IL-2Rα/CD25 have reached clinical trials. NKTR-214 is composed of recombinant IL-2 together with multiple molecules of PEG. Directed PEGylation generates an inactive cytokine with a long half-life in circulation. The PEG groups are progressively released, yielding IL-2 molecules with double or single PEGylation that can interact with the medium-affinity IL-2 receptor but not with the high-affinity IL-2 receptor.^33^ This modified cytokine is being evaluated in clinical trials in combination with the immune checkpoint inhibitors atezolizumab (NCT03138889), nivolumab (NCT02983045, NCT03282344 and NCT03435640) and nivolumab plus ipilimumab (NCT02983045). As reported at the American Society of Clinical Oncology (ASCO) annual meeting in 2018, NKTR-214 has undergone dose-escalation studies and has also been used in combination with nivolumab to treat 214 patients, showing promising response rates in immunotherapy-naive patients suffering from melanoma, RCC or NSCLC. In terms of safety, the combination was tolerated, and comparative randomised studies will be performed to confirm benefit over nivolumab single-agent therapy.^34^

Another strategy to avoid signalling through the high-affinity receptor uses an engineered mutated IL-2 variant that shows reduced binding to IL-2Rα. This mutant is fused to antibodies to target the cytokine to the TME. In the case of cergutuzumab amunaleukin, the IL-2 variant is fused to an antibody that targets it to the carcinoembryonic antigen.^35^ A phase I clinical trial is evaluating this fusion protein in combination with atezolizumab (NCT02350673). In the case of RO6874281, the mutated cytokine is fused to an antibody that targets the fibroblast activation protein which is expressed by cancer-associated fibroblasts. This fusion protein is being combined in clinical trials with the epidermal growth factor receptor inhibitors trastuzumab or cetuximab (NCT02627274), with atezolizumab (NCT03386721 and NCT03063762) as well as with bevacizumab, a monoclonal antibody that targets vascular endothelial growth factor (VEGF) (NCT03063762).

### Interleukin-15

IL-15 is mainly produced by activated myeloid cells as a membrane-bound heterodimer associated with IL-15Rα in such a way that it is trans-presented to NK cells and T cells expressing IL-2/IL-15Rβ and the common γ chain receptor.^36,37^ Importantly, IL-15 is critically needed for the ontogeny of NK cells and CD8^+^ T cells^38,39^ and also induces the proliferation, cytotoxic action and the release of other cytokines such as IFN-γ^38,40^ from these cells, highlighting its role in potentiating the immune response. Preclinical observations strongly support the potential anti-tumour activity of IL-15 mediated by NK cells and T lymphocytes.^41,42^ Unlike IL-2, IL-15 does not stimulate Treg cells, a subset that might reduce the anti-tumour activity of NK and T cells. This is due to the fact that IL-15 does not bind to the IL-2Rα chain (also known as CD25).^43^

First-in-human clinical trials using recombinant aglycosylated IL-15 produced in *Escherichia coli* consisted of intravenous bolus administration to patients with advanced melanoma or RCC. Patients showed an expansion of NK and CD8^+^ T cells in peripheral blood, but there were severe adverse events and dose-limiting toxicities, including high fever, hypotension and thrombocytopenia.^44^ This trial stopped due to dose-limiting toxicity at 1.0 μg/kg per day. There were no clinical responses by Response Evaluation Criteria In Solid Tumours (RECIST) v1.1 criteria, but 5 out of 18 patients with malignant melanoma or RCC showed a reduction of between 10% and 30% in their marker lesion. In another clinical trial reported recently, IL-15 was subcutaneously administered to patients with melanoma, RCC, non-small cell lung cancer (NSCLC) or squamous cell head and neck carcinoma.^45^ The maximum tolerated dose of IL-15 administered subcutaneously was significantly higher than the dose that was feasible by intravenous bolus injection, and reached 3.0 μg/kg per day. The number of circulating NK cells dramatically increased with IL-15 administration in a dose-dependent fashion. There were no objective clinical responses in this trial, but several patients had disease stabilisation, including a patient with RCC whose disease was stable for over 2 years.^45^

**Fig. 2:**
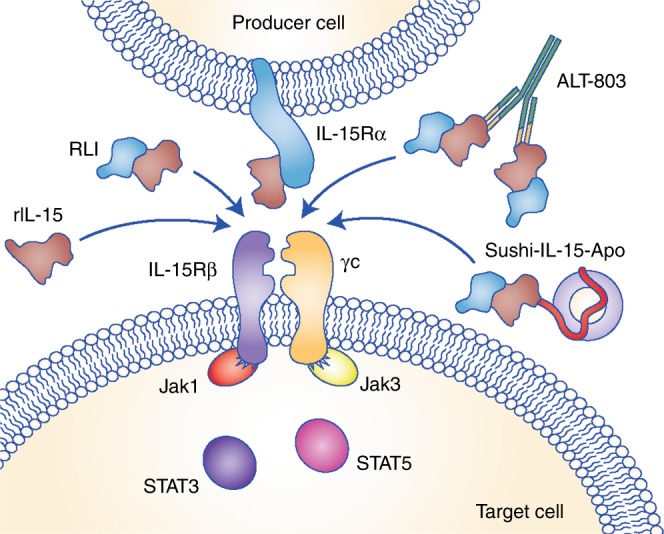
Engineered interleukin-15 (IL-15) variants. IL-15 is mainly produced as a membrane-bound heterodimer associated with IL-15Rα. The interaction of the IL-15–IL15-Rα complex with IL-2/IL-15Rβ and the common γc receptor triggers the phosphorylation of Janus kinase-1 (JAK1) and JAK3 and the recruitment and subsequent phosphorylation of signal transducer and activator of transcription-3 (STAT3) and STAT5. Clinical trials have tested the safety and efficacy of recombinant aglycosylated IL-15 and engineered variants to mimic the trans-presentation process and to enhance the half-life in circulation. These variants included the superagonist RLI protein (comprising the binding domain of IL-15Rα fused to IL-15), ALT-803 (comprising mutated IL-15 fused to the binding domain of IL-15Rα and an IgG1 Fc domain) and Sushi-IL15-Apo (a fusion protein encompassing the binding domain of IL-15Rα, IL-15 and apolipoprotein A-I)

In vitro and in vivo preclinical studies indicate that IL-15 is more bioactive when trans-presented adsorbed onto the IL-15Rα receptor subunit.^46–48^ Recombinant IL-15 is quickly eliminated from the blood due to its small molecular size (it has an in vivo half-life of 2.5 h), and several approaches have therefore focused on designing more stable protein constructs encompassing IL-15 and IL-15Rα that display a longer half-life and better biodistribution parameters. The different therapeutic forms of IL-15 and its signalling receptor are described below and schematically represented in Fig. 2.

The recombinant protein RLI encompasses the binding domain of IL-15Rα (the so-called sushi domain) bound to IL-15 by a flexible linker; this fusion protein displays superagonistic activity towards the IL-15R–β/γ complex and exerts anti-tumour properties in in vivo models.^49^ Some variations of the original structure have been made to redirect IL-15 to the tumour, such as the addition of anti-CD20 or anti-GD2 antibody domains.^50,51^ Our group has constructed a recombinant protein termed Sushi-IL15-Apo, a triple fusion protein comprising human IL-15, the binding domain of IL-15Rα and apolipoprotein A-I. Apolipoprotein A-I, the major component of high-density lipoproteins, binds to its main receptor, SR-BI, which is overexpressed on liver and tumour cells, thereby effectively targeting IL-15 activity within the recombinant protein to the tumour.^52–54^

ALT-803, another variant of IL-15 that encompasses the sushi domain and a mutated IL-15 fused to an IgG1 Fc domain, has been shown to promote strong IL-15 activity in mouse models^55^ and has been tested in clinical trials. In the dose-escalation phase I clinical study with this recombinant protein, 33 patients with haematological cancer received ALT-803 after disease relapse following bone marrow allotransplantation.^56^ Sixteen subjects received the treatment intravenously and 17 received it subcutaneously. The treatment was well tolerated in both cohorts. All patients showed an increase in the levels of circulating NK and CD8^+^ T cells (the increase was more pronounced in the subcutaneous treatment cohort), and 19% of the subjects met criteria for clinical benefit. Another trial has been performed with ALT-803 in 21 patients with metastatic NSCLC, testing escalating doses via subcutaneous administration in combination with anti-PD-1 therapy. The recombinant protein drug was well tolerated, no dose-limiting toxicities were recorded and 29% of patients achieved an objective response. A phase II continuation clinical trial is ongoing (NCT02989844).^57^ It has also been reported that ALT-803 is well tolerated in combination with intravesical bacillus Calmette-Guérin therapy for high-risk localised bladder cancer, achieving sustained complete responses for 24 months in 9 out of 9 patients.^58^ These data have led the FDA to grant fast-track designation to this treatment for patients with non-muscle invasive bladder cancer.

Other clinical studies involving IL-15 proteins as experimental agents are ongoing. Most also include administering IL-15 in combination with other agents. Some trials are testing IL-15 as an adjuvant in T cell or NK cell adoptive cell therapies (NCT01875601, NCT02465957 and NCT01385423). Other clinical studies have been designed to test the ability of IL-15 to increase antibody-dependent cellular cytotoxicity (ADCC) effects, using IL-15 in combination with tumour-targeting monoclonal antibodies such as alemtuzumab (NCT02689453) or rituximab (NCT02384954).

### Interleukin-21

IL-21 is another cytokine from the IL-2-family that is also being actively tested in cancer clinical trials alone (NCT00095108, NCT00514085 and NCT00336987) or in combination with ipilimumab (NCT01489059), nivolumab (NCT01629758), sunitinib (NCT00617253), rituximab (NCT00347971), sorafenib (NCT00389285) or doxorubicin (NCT00523380). In a dose-escalation phase I trial, there were pharmacodynamic effects of anti-tumour immunity upregulation, and 3 out of 26 patients experienced objective partial responses.^59^ In non-Hodgkin lymphoma patients, recombinant IL-21 has been tested in combination with rituximab, achieving clinical responses in 8 out of 19 patients.^60^ There is also interest in using IL-21 to potentiate adoptive T cell therapy.^61^ However, the clinical development of this cytokine is still in its infancy and progress is likely to occur in the form of combinations.

### Interleukin-10

IL-10 is released by innate and adaptive immune cells to fine-tune the activity of pro-inflammatory cytokines.^62^ IL-10 is considered to be an immunosuppressive cytokine as it can decrease the antigen-presenting activity of DCs^63^ and inhibit the cytotoxic and cytokine-release functions performed by T and NK lymphocytes.^64^ However, recent reports point to a context-dependent outcome of IL-10 activity.^65^ In chronic infections and cancer, autocrine IL-10 activity on CD8^+^ T lymphocytes has been shown to be crucial for inhibiting antigen-induced CD8^+^ T cell apoptosis, thereby prolonging the effector activity of these cytotoxic lymphocytes.^66,67^ This concept has been evaluated in a phase I clinical trial in advanced, treatment-refractory tumours (NCT02009449) using IL-10 conjugated with PEG to increase its half-life. Administration of the PEGylated cytokine (termed pegilodecakin) is well tolerated, and grade 3–4 immune-related adverse effects were detected in only 15% of patients. Partial responses were observed in patients with uveal melanoma, RCC and colorectal cancer.^68^ At ASCO 2018, it was reported that the combination of pegilodecakin with nivolumab or pembrolizumab was tolerable in 38 patients with RCC. The clinical activity of pegilodecakin looks promising, with 50% of patients achieving a RECIST 1.1 response, 9% of which were complete.^69^

### Interleukin-12

The IL-12 family comprises unique heterodimeric cytokines that include IL-12, IL-23, IL-27 and IL-35. The heterodimeric 70 kDa biologically active form of IL-12 (p35–p40) is composed of two independently produced subunits, α (IL12p35) and β (IL12p40), linked by disulphide bonds. The α subunit (IL12p35) shares sequence homology with IL-6 and can also be part of IL-23 (p19–p40), whereas the β subunit (IL12p40) is shared with IL-35 (p35/Ebi3).^70^

IL-12 is mainly produced by activated antigen-presenting cells such as DCs, macrophages, monocytes and B cells. IL-12 production is a tightly controlled process, regulated mainly at the transcriptional level. Production is initiated by activation of pathogen recognition receptors such as Toll-like receptors in antigen-presenting cells upon sensing of pathogen-associated molecular patterns) or damage-associated molecular patterns.^71^ In addition, cytokine stimulation and direct immune cell–cell contact, including CD40–CD40L interactions, induce IL-12 production. This latter CD40-dependent mechanism is likely to be the principal mechanism for the production of IL-12 in cancer.^72^ IL-12 binds to the IL-12 receptor (IL-12R), expressed as a high-affinity heterodimer of IL-12Rβ1 and IL-12Rβ2 subunits mostly on T cells and NK cells. The β1 subunit is constitutively expressed on immune cells, whereas the β2 subunit is upregulated in T cells and NK cells upon activation.^73^

IL-12R activation results in the recruitment of the kinases Janus kinase (JAK2) and Tyk2 and subsequent phosphorylation of signal transducer and activator of transcription 4 (STAT4). Dimerisation and nuclear translocation of phosphorylated STAT4 ultimately leads to IFN-γ production, transcriptional reprogramming of CD4^+^ and CD8^+^ T cells towards type 1 T helper (Th1) cell differentiation and maturation of NK cells. IFN-γ causes a FAS-ligand-dependent collapse of the myeloid compartment and M1 macrophage polarisation, paving the way for an optimal T cell infiltration and cytolytic function.^74,75^ Furthermore, the potent antiangiogenic properties of IL-12 also render it an attractive cytokine for cancer treatment.^71,76^ The antiangiogenic effects are mediated by the production of IFN-γ which alters extracellular matrix remodelling and the expression of adhesion molecules on endothelial cells.^77^ An interesting antiangiogenic loop involving IL-12, IFN-γ and CXC chemokine ligand 10 (CXCL10) has been discovered and involves CXCR4 expression on proliferating vascular endothelial cells.^78^

In preclinical studies, systemic administration of recombinant IL-12 induced potent anti-tumour efficacy in xenograft mouse models.^75,79^ However, in clinical trials, the short half-life of the recombinant protein in serum meant that high and multiple doses were required, which resulted in dose-related toxicities, mainly caused by elevated levels of circulating IFN-γ.^80^ To overcome the toxicities, different approaches have been proposed, focusing on local delivery of IL-12 to avoid toxic systemic exposure. Several preclinical studies exploiting local gene transfer using viral vectors,^81^ liposomes^82^ or in vivo electroporation of an IL-12-encoding plasmid in accessible tumour lesions^83^ have demonstrated strong anti-tumour activity with no apparent toxicity. Focusing on targeted protein delivery, a fusion protein encompassing single-chain IL-12 coupled to an extracellular double-stranded-DNA binding antibody (which thereby targets tumour necrotic areas) showed enhanced anti-tumour activity in mouse models.^84^

IL-12-based local treatment can be synergistically combined with adoptive T cell transfer. In relation to this approach, the use of transgenic tumour-specific T cells engineered to secrete IL-12 in the TME in response to TCR-mediated antigen stimulation was evaluated in mouse models, and showed very promising efficacy.^85^ However, leakage during production of the inducible IL-12 by the adoptively transferred TILs was reported in a subsequent clinical trial, leading to unacceptable toxicity, with one lethal case.^86^

Local IL-12 delivery and the use of immunostimulatory monoclonal antibodies can generate synergistic results. In line with this, combining the local delivery of IL-12 with the blockade of PD-1–PD-L1 has been shown to eradicate large established tumours in preclinical models.^81^ An in vivo electroporation approach is currently undergoing clinical trials for local delivery of IL-12 as a single agent (NCT01579318, NCT00323206, NCT01502293 and NCT02345330)^87^ and in combination with pembrolizumab (NTC02493361 and NTC03132675).

### Granulocyte-macrophage colony-stimulating factor

GM-CSF (CSF-2) is a clinically available recombinant cytokine used to promote myeloid reconstitution after bone marrow transplantation or following induction chemotherapy in patients with acute myelogenous leukaemia. This cytokine promotes the expansion and activation of myeloid cells such as DCs and macrophages. For this reason, it provides an adjuvant effect for various types of vaccines. As a serious drawback, however, GM-CSF also promotes the differentiation and accumulation of tumour-associated myeloid cells which support tumour growth. Subcutaneous recombinant GM-CSF given in combination with ipilimumab improved the overall survival and reduced ipilimumab-related toxicity in patients with advanced melanoma.^88^ The adjuvant activity of GM-CSF is exploited in the FDA-approved drug talimogene laherparepvec (T-VEC). This genetically modified herpes simplex virus encodes GM-CSF and, upon intratumoural injection, has demonstrated overall response benefit over subcutaneous GM-CSF in advanced melanoma patients.^89^ GM-CSF gene transfection into autologous or allogeneic tumour cells has been the basis for GVAX products which, in the allogeneic setting, have not met satisfactory endpoints in prostate cancer phase III trials, but hold promise in the autologous setting and in combination regimens in pancreatic cancer.^90,91^

## Inhibiting the immunosuppressive activity

Cytokines might be released or activated in the TME by tumour cells or infiltrating immune cells to promote all phases of tumourigenesis. In this case, strategies to neutralise the pathogenic activity of such cytokines can be developed to enhance cancer immunotherapy. These strategies not only include the use of antagonistic antibodies but also polypeptides, cytokine traps, small interfering RNA (siRNA) and small molecules that inhibit signal transduction from cytokine receptors. However, it must be kept in mind that, like double-edged swords, some of these cytokines may exert pro- and anti-tumour activities, depending on context and elements in the TME.

### TNF-α

TNF-α is a pro-inflammatory cytokine produced mainly by myeloid-derived cells such as monocytes, macrophages and DCs, although many other cells such as T lymphocytes, endothelial cells, adipocytes and fibroblasts can also produce this cytokine under stressful conditions. TNF-α is recognised by two receptors with a broad tissue distribution: TNFR1 and TNFR2.^92^

TNF-α is a key pathogenic mediator of several autoimmune diseases such as rheumatoid arthritis, Crohn’s disease, ulcerative colitis, psoriasis, psoriatic arthritis and ankylosing spondylitis. TNF-α activates macrophages at the inflammation site inducing the release of other pro-inflammatory cytokines that exacerbate inflammation. The activity of TNF-α on epithelial cells impairs barrier function and promotes permeability to commensal bacteria. The activity of TNF-α on fibroblasts leads to the expression of metalloproteinases and the synthesis of collagen, thereby promoting tissue fibrosis. Finally, TNF-α acts on endothelial cells to increase the adhesion molecules expressed on blood vessels, subsequently leading to increases in leucocyte infiltration; it might also induce endothelial cell apoptosis.

The relevance of this cytokine in autoimmune diseases led to the generation and approval of several TNF-α antagonists such as infliximab, adalimumab and etanercept.^92^ Infliximab is also included in the guidelines for the treatment of several autoimmune-like syndromes that are associated with immune checkpoint inhibitor treatment that are refractory to corticosteroid treatment.^11^

In cancer immunotherapy, TNF-α is mainly considered as a mediator of anti-tumour immune responses, and several immunotherapies have shown depleted anti-tumour efficacy when co-administered with TNF-α antagonists.^93,94^ It is likely that acute immune responses are boosted by TNF-α release. However, chronic exposure to TNF-α can promote tumour growth by mediating activation-induced cell death of effector T lymphocytes.^95^ In mouse models, TNF-α has been shown to have a detrimental effect on immunotherapies based on the blockade of the PD-1 pathway. The proposed mechanism involves the TNF-α-mediated upregulation of the secondary checkpoint component TIM-3 in CD8^+^ T lymphocytes, induced by anti-PD-1 antibody therapy.^96^ Safety of the triple combination of ipilimumab, nivolumab and an antibody to block TNF-α (infliximab or certolizumab) is being evaluated in a phase I clinical trial (NCT03293784).

### TGF-β

TGF-β plays a dual role in the tumourigenic process. During the initial stages of tumourigenesis, TGF-β inhibits tumour development due to cell-cycle blockade in cells undergoing transformation. However, tumour cells develop resistance mechanisms to the anti-proliferative activity of TGF-β,^97^ and in later stages of tumour development, TGF-β acts both on tumour cells and on cells of the TME to promote tumour progression. In tumour cells, TGF-β is a key mediator of the epithelial–mesenchymal transition. In the tumour stromal compartments, this cytokine promotes the release of angiogenic factors, such as VEGF, and the recruitment of Treg cells and myeloid cells with a pro-tumour polarisation, such as neutrophils, macrophages, myeloid-derived suppressor cells (MDSCs) and tolerogenic DCs. Moreover, TGF-β decreases the activity of NK cells and CD8^+^ T lymphocytes.^98^

The relevance of the immunosuppressive activity of this cytokine in tumours has been conducive to the development of several small molecules, peptides, antisense oligonucleotides, cytokine traps and antibodies to block the TGF-β pathway in cancer.^98^ Although their efficacy as monotherapy agents has been disappointing, their activity in combination with anti-PD-1 or anti-PD-L1 agents has renewed interest in inhibitors tackling the functions of this cytokine. TGF-β might be the main driver of immune cell exclusion in several tumours.^99^ Numerous clinical trials are testing the safety and anti-tumour activity of the combined blockade of TGF-β using small molecules, such as galunisertib, or antagonistic monoclonal antibodies, such as fresolimumab, with PD-1–PD-L1 such as nivolumab or durvalumab (NCT02423343 and NCT02734160). Additionally, M7824, a compound designed to simultaneously block both of these targets, is undergoing clinical testing (NCT03451773 and NCT03451773). This construct combines a chimeric version of an anti-PD-L1 monoclonal antibody (avelumab) with a fragment of TGF-βR4 to entrap active TGF-β.^100^ Inhibiting this cytokine could be especially relevant in tumours treated with radiotherapy, as radiotherapy activates the latent form of TGF-β and induces its transcription.^101,102^

### CSF-1 and other cytokines that promote myeloid cells

Immune cells differentiated from haematopoietic myeloid precursors are key components of the immune response. The myeloid cells that can be found in tumours are tolerogenic DCs, tumour-associated macrophages (TAMs), tumour-associated neutrophils and MDSCs, all of which comprise a heterogenous population of immature myeloid precursors arrested in their differentiation process.^76,103^

Tumour-released factors promote the expansion, recruitment and polarisation of these myeloid cell populations, which reciprocally support tumour development through the release of anti-inflammatory cytokines, pro-angiogenic mediators and growth factors. Furthermore, these cells express immune checkpoint ligands such as PD-L1 on their surface, as well as enzymes that promote metabolic alterations in the TME. All these mechanisms reduce the release of cytokines by, and cytotoxic activity of, NK and T lymphocytes. Strategies to counteract these tumour-promoting activities involve depleting or repolarising myeloid cells to restore efficient activity of anti-tumour effector immune cells such as NK cells, Th1 CD4^+^ T lymphocytes and CD8^+^ cytotoxic T lymphocytes, as well as their infiltration into the malignant tissue.^76^

CSF-1 receptor, the receptor for CSF-1 and IL-34, is expressed on TAMs and maintains the homoeostasis of myeloid cells. By contrast, M1 macrophages with anti-tumour activity are not dependent on the CSF-1 receptor, making this pathway an excellent target for small molecules, such as pexidartinib, or monoclonal antibodies, such as cabiralizumab. CSF-1R blockade leads to the alteration of macrophage polarisation in the TME, which shows lower myeloid cell content associated with a certain degree of tumour regression in preclinical animal models.^104,105^

The accumulation of myeloid cells in the TME is orchestrated by chemokine gradients. The most relevant cytokines in myeloid cell recruitment are CXCL8 (also known as IL-8), CC chemokine ligand 2 (CCL2), CCL3 and CCL5.^106^ CXCL8/IL-8 is produced by tumour cells, and elevations in the serum concentrations of this chemokine reflect tumour growth dynamics and have been proposed as a biomarker that can predict the response to anti-PD-1 monoclonal antibodies in melanoma and NSCLC.^107^ An antibody to block the interaction of CXCL8/IL-8 with its receptors on myeloid cells, CXCR1 and CXCR2, has been tested in a phase I clinical trial in patients with metastatic or unresectable, locally advanced stage solid tumours (NCT02536469) and is now entering into a phase Ia/II study in combination with nivolumab (NCT03400332).^108,109^

CCR2 is the receptor for CCL2, while CCR5 binds both to CCL3 and CCL5. CCR2/CCR5 inhibitors are being developed to synergistically block the activity of these chemokines, thereby inhibiting myeloid cell recruitment, in combination with either chemotherapy or nivolumab in patients with metastatic colorectal cancer or pancreatic cancer (NCT03184870). Both CCR2 and CCR5 are involved in the recruitment of Treg cells, TAMs and MDSCs. CCR2 is also involved in the bone marrow egress of myeloid cells. In addition, CCR5 blockade can repolarise TAMs from a pro-tumourigenic phenotype to an M1 phenotype.^110^

Finally, several small molecules and monoclonal antibodies targeting the VEGF pathway have been approved for the treatment of several types of cancer. These compounds were originally developed to interfere with the angiogenic activity of VEGF-A, but it is now known that this pleiotropic cytokine is also involved in the recruitment of immunosuppressive myeloid cells to the TME.^111^ The relevance of this concept in the clinic has been highlighted by the results of the phase II study of atezolizumab with or without bevacizumab in patients with untreated metastatic RCC. Patients who showed tumour immune signatures of T effector cells and myeloid cell infiltration were refractory to treatment with atezolizumab alone but responded to the combination of bevacizumab and atezolizumab.^112^ Moreover, a combination of bevacizumab plus atezolizumab plus chemotherapy provides evidence for activity against NSCLC as reported in ASCO 2018.^113^ The combination of bevacizumab and atezolizumab has also been tested in a phase I trial for advanced hepatocellular carcinoma patients, showing good tolerability and a very promising 62% overall response rate.^114^

## Conclusions

Cytokines are potent but complex immune mediators. Making cytokine-based drugs is a formidable challenge that requires a profound knowledge of cytokine biology and contemporary biotechnology to exploit their anti-tumour activity while keeping toxicity to a minimum. The approved monoclonal antibodies against the PD-1–PD-L1 axis provide a tool to reinvigorate effector lymphocytes but primary and acquired resistance mechanisms limit the fraction of patients who benefit from these novel immunotherapies. Cytokines will be key molecules to overcome such resistance mechanisms due to their ability to expand and reactivate effector NK and T lymphocytes and promote tumour infiltration by lymphocytes as well as their persistence in the TME. Current excitement surrounding the immunobiology of cytokines has resulted in a number of ongoing clinical trials (Table 1), the results of which are eagerly expected. The possibility of blocking cytokines and chemokines that mediate the recruitment of Treg cells and tumour-associated myeloid cells might expose an as yet under exploited Achilles’ heel in tumours, against which we can deploy promising combination strategies.

**Table 1:** Representative cytokine clinical trials

Cytokine	Agent	Main mode of action	Clinical trial
IL-15	ALT-803 IL-15+T or NK cells IL15+alemtuzumab IL15+rituximab	Expansion of NK and T lymphocytes	NCT02989844 NCT01875601 NCT02465957 NCT01385423 NCT1369888 NCT02689453 NCT02384954
IL-2	NKTR-214+atezolizumab NKTR-214+nivolumab NKTR-214+nivolumab+ipilimumab Cergutuzumab amunaleukin+atezolizumab RO6874281+trastuzumab or cetuximab RO6874281+atezolizumab RO6874281+atezolizumab+bevacizumab	Expansion of NK and T lymphocytes	NCT03138889 NCT02983045 NCT03282344 NCT03435640 NCT02983045 NCT02350673 NCT02627274 NCT03386721 NCT03063762 NCT03063762
TNF-α	Nivolumab+ipilimumab+certolizumab or infliximab	Blockade of activation-immune cell death of tumour-infiltrating lymphocytes	NCT03293784
IL-10	Pegilodecakin+FOLFOX	Blockade of activation-immune cell death of tumour-infiltrating lymphocytes	NCT02923921
IL-12	Transduced TILs Electroporated plasmid Electroporated plasmid+pembrolizumab	Promotion of NK cells and Th1 CD4^+^ and CD8^+^ lymphocytes	NCT01236573 NCT01579318 NCT00323206 NCT01502293 NCT02345330 NCT02493361 NCT03132675
TGF-β	Galunisertib+nivolumab Galunisertib+durvalumab Fresolimumab+radiotherapy M7824 (dual block of TGF-β and PD-L1)	Inflammation of immune-excluded tumours	NCT02423343 NCT02734160 NCT02581787 NCT03451773 NCT03451773
CSF-1	Cabiralizumab+nivolumab Pexidartinib+durvalumab Pexidartinib+durvalumab or tremelimumab Pexidartinib+pembrolizumab	Suppression of tumour-associated myeloid cell	NCT03502330 NCT02777710 NCT02718911 NCT02452424
IL-8	mAb anti-IL-8+nivolumab	Suppression of tumour-associated myeloid cell	NCT03400332
CCL2, CCL3 and CCL5	CCR2/CCR5 inhibitor+nivolumab or chemotherapy	Suppression of tumour-associated myeloid cell	NCT03184870
VEGF	Bevacizumab+atezolizumab	Suppression of tumour-associated myeloid cell	NCT01984242

**Fig. 3:**
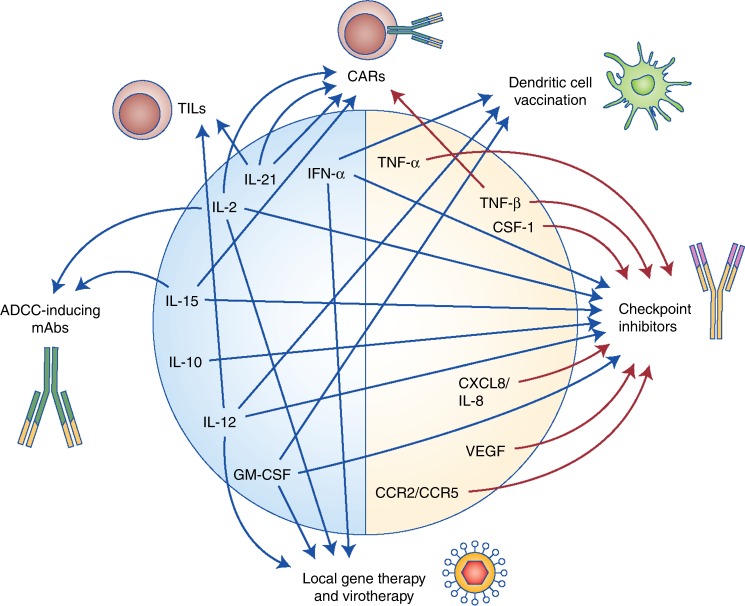
Potential combinations of cytokine-based drugs with other modalities of cancer immunotherapy. Cytokines have been dichotomised into immunostimulatory cytokines (blue) and immunosuppressive cytokines (beige). The arrows indicate combinations with other immunotherapy approaches that are currently under research. TILs tumour-infiltrating lymphocytes, CARs chimeric antigen receptor T cells, ADCC antibody-dependent cellular cytotoxicity

In future developments, two key aspects are to be considered: confining the effects of the cytokines to the site of action to avoid systemic pro-inflammatory effects, and including these treatments in combination immunotherapy strategies.^15^ For the first point, approaches based on targeting agents to the TME or intratumoural administration of the proteins or their encoding genes can be envisioned. These tumour-targeted approaches would also be relevant for the neutralisation of immunosuppressive cytokines. Cytokines in the realms of gene therapy, cell therapy and monoclonal antibody-based therapies might become formidable partners in elegant synergistic strategies (Fig. 3), whose anti-tumour efficacy only the future will reveal.
